# Nutritional iodine intake in patients with anorexia nervosa

**DOI:** 10.1530/ETJ-25-0076

**Published:** 2025-09-19

**Authors:** Sophie Seydoux, Mathias Halbout, Sandra Gebhard, Michael B Zimmerman, Peter A Kopp

**Affiliations:** ^1^ Faculty of Biology and Medicine, University of Lausanne, Lausanne, Switzerland; ^2^ Division of Endocrinology, Diabetes and Metabolism, University Hospital of Lausanne and University of Lausanne, Lausanne, Switzerland; ^3^ Department of Psychiatry, Centre Vaudois Anorexie Boulimie, University Hospital of Lausanne, Lausanne, Switzerland; ^4^ Human Nutrition Laboratory, Institute of Food, Nutrition, and Health, Swiss Federal Institute of Technology Zürich, Zürich, Switzerland

**Keywords:** iodine, thyroid hormones, anorexia nervosa, hypothyroidism, non-thyroidal illness

## Abstract

**Objective:**

Iodine deficiency (ID) causes a wide range of health issues, from endemic goiter to more subtle effects resulting from reduced thyroid hormone production. The recommended daily iodine intake for adolescents and adults is 150 μg, which corresponds to a median urinary iodine concentration (UIC) of 100–299 μg/L at the population level. Individuals with anorexia nervosa typically suffer from deficiencies in micronutrients and vitamins, but there is little data on iodine status. This study assessed UIC and associated factors in a cohort of patients with anorexia nervosa.

**Methods:**

This was a prospective monocentric exploratory observational study performed at the Centre Vaudois anorexie boulimie (abC) and the Division interdisciplinaire de santé des adolescents (DISA) of the Centre Hospitalier Universitaire Vaudois, University of Lausanne, Switzerland. The study included 39 patients with anorexia nervosa, aged ≥14 years, recruited between May and August 2022. After obtaining informed consent, anthropometric data were extracted from the electronic medical record and random spot urine samples were collected. The UIC was determined by ion-chromatography mass spectrometry.

**Results:**

Median age (IQR) was 18 (14–62) years and median body mass index (BMI) was 17.72 (14.86–23.54) kg/m^2^. Median UIC was 67.7 μg/L, and 22/39 individuals had a UIC <100 μg/L. There was a positive correlation between BMI and UIC (*P* = 0.047).

**Conclusion:**

The findings suggest that patients with anorexia nervosa are at risk of ID, and lower BMI predicts lower UIC. Although these data need to be corroborated in a larger cohort, clinicians caring for patients with anorexia should consider recommending an iodine-containing multivitamin.

## Introduction

Iodine is an essential component for the synthesis of the thyroid hormones thyroxine (T4) and triiodothyronine (T3), which play a preeminent role in human development, growth, and metabolism (1). Normal levels of thyroid hormones are crucial for intrauterine growth and neurocognitive development of the fetus, neuronal migration and myelination, and for normal metabolism (2, 3, 4, 5, 6). Historically, iodine deficiency disorders (IDD) have been a major global health problem resulting in endemic goiter and cretinism, i.e., mental retardation secondary to intrauterine and/or postnatal hypothyroidism (7, 8). However, severe IDD have now been largely eradicated on a global scale through the introduction of salt iodization (9, 10). In 2022, close to 90% of the global population had access to iodized salt, and most countries have an adequate iodine intake (11).

Iodine is found in foods such as fish and eggs, but the most important source consists of the iodized salt in processed foods (12). According to the World Health Organization (WHO), adequate nutritional iodine intake for school-age children is 90 μg per day and 150 μg for adolescents and adults (9, 13). This is associated with a median urinary iodine concentration (UIC) in the population of 100–299 μg/L (13). Pregnant and lactating women need a higher daily iodine intake of about 250 μg iodine per day, associated with a UIC of 150–499 μg/L (5, 14). The WHO defines iodine deficiency (ID) in a population as a median UIC ≤100 μg/L in adults and children, and ≤150 μg/L in pregnant and lactating women (9, 13). The introduction of iodized salt has had major positive effects on the prevalence of goiter and hypothyroidism, thyroid size, and the average intelligence quotient, and it eliminates intellectual disabilities due to impaired synthesis of thyroid hormones (3, 15).

Children and pregnant women are not the only populations that are particularly vulnerable to ID. Individuals with poor dietary intake and/or malnutrition can also develop ID (12), and this might also include individuals with anorexia nervosa. However, there is little data on iodine intake in patients with anorexia nervosa, and previous reports are limited to individual case studies of iodine-induced hypothyroidism (16, 17, 18). The consequences of anorexia nervosa are multiple and include psychiatric manifestations, endocrine dysregulation with hypothalamic hypogonadism, low bone mineral density, and a spectrum of nutritional deficiencies (19, 20). Many of these patients also have alterations in the hypothalamic–pituitary–thyroid axis consistent with a ‘low T3 syndrome’. The biochemical profile includes low T3 levels, with thyroid stimulating hormone (TSH) levels that remain typically within the reference range (20, 21).

We hypothesized that ID may be part of the multiple deficiencies in micronutrients and vitamins affecting patients with anorexia nervosa. Therefore, the primary objective of this study was to prospectively determine the urinary iodine status in a cohort of patients with anorexia nervosa.

## Materials and methods

### Study design and participants

The study was approved by the ethics committee of the Canton de Vaud (Commission cantonale d'éthique de la recherche sur l'être humain (CER-VD), protocol number 2021-02126). All participants gave informed written consent; for patients between the age of 14 and 18, assent was also obtained from the parents.

Patients were recruited prospectively at the Centre Vaudois anorexie boulimie (abC) (Center for anorexia and bulimia) and the Division interdisciplinaire de santé des adolescents (DISA) (Interdisciplinary division for adolescent health) of the University Hospital (Centre Hospitalier Universitaire Vaudois) at the University of Lausanne, Switzerland.

Eligible participants were patients with anorexia nervosa as defined by ICD-10 diagnostic criteria, aged ≥14 years, at different stages of their treatment. All data including age, sex, anthropometric data (height, weight, body mass index (BMI)), medication, and medical history were collected in a case report form (Appendix I (see section on Supplementary materials given at the end of the article)).

Two subgroups were established based on the reported eating behavior: restrictive or binge eating/purging anorexia nervosa. The former is defined by severe limitation of food intake leading to undernutrition, frequent avoidance of certain food groups, obsessive eating rules, and, in some patients, excessive exercise. In the binge eating/purging form, these eating behaviors are further complicated by episodes of binge eating and/or purging behaviors such as self-induced vomiting or misuse of laxatives or diuretics.

### Urinary iodine measurement

A random urine sample was collected from all participants and kept frozen at −20°C until analysis. The samples were coded to assure confidentiality. The UIC, the accepted biomarker characterizing the dietary iodine intake at the population level, was determined by ion-chromatography mass spectrometry in the Human Nutrition Laboratory of Prof. Michael Zimmermann at the Swiss Federal Institute of Technology Zürich (Eidgenössiche Technische Hochschule Zürich (ETHZ)). Duplicate measurements were performed for every urine sample, and the mean was calculated for further analysis.

If available, thyroid function tests (TSH; T4; T3) were extracted from the electronic medical record.

### Statistical analysis

Normality for the UIC data was tested with the Shapiro–Wilk test. Because of a significant departure from normality, the descriptive statistics are presented as median (± standard deviation), minimum, and maximum.

The distribution of the individual quantitative UIC data was summarized in a histogram, including the cutoffs for iodine sufficiency in adults and children (≥100 μg/L), and pregnant women (≥150 μg/L) at the population level.

Comparisons between patients with a BMI <18.5 kg/m^2^ and >18.5 kg/m^2^, restrictive and purging anorexia, and their respective UIC were made with the Mann–Whitney test for independent samples.

## Results

### Demographics and clinical presentation

In total, 40 patients were enrolled into the study between May 2022 and August 2022. One patient was excluded because of an extremely high UIC of 28,305 μg/L; the reason for this finding was an angiography computerized tomography scan in the days preceding the urine collection.

The baseline characteristics of the 39 enrolled patients are summarized in Table 1. Of the patients, 38 (97.4%) were female, and the median age was 18 years. The median weight was 48.85 kg, and the median BMI was 17.72 kg/m^2^. Of the 39 patients, 11 (28.2%) were classified as having binge eating/purging anorexia nervosa, and 28 (71.8%) patients had restrictive anorexia nervosa.

**Table 1:** Baseline characteristics of the study population (*n* = 39). Data are presented as median (IQR) or as *n* (%).

Characteristics	Values
Age, years	18 (14–62)
Female	38 (97.4%)
Weight, kg	48.85 (39.0–66.45)
Height, cm	164.00 (151.50–178.00)
BMI, kg/m^2^	17.72 (14.86–23.54)
Type of anorexia nervosa	
Restrictive	28 (71.8%)
Binge eating/purgating	11 (28.2%)

IQR, interquartile range; BMI, body mass index.

### Urinary iodine concentration

**Figure 1:**
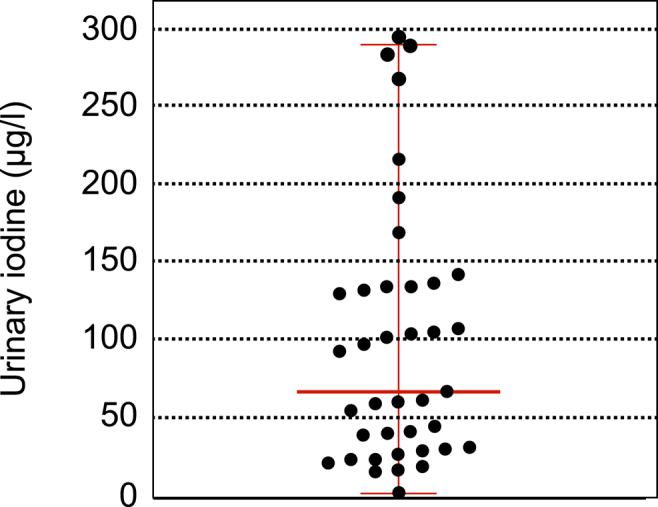
Whisker plot of the urine iodine concentration (UIC) data with median, minimum, and maximum.

**Figure 2:**
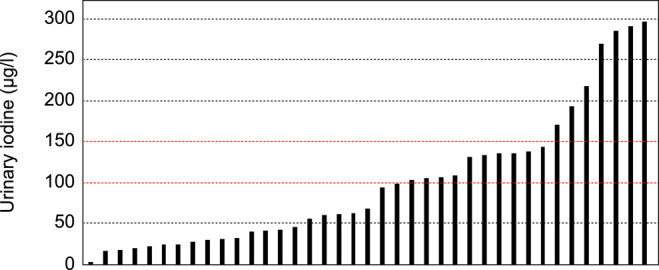
Histogram with the individual quantitative UIC data of the 39 participants. At the population level, a median UIC below 100 μg/L is indicative of an insufficient iodine intake in adolescents and adults. For pregnant and lactating women, the cutoff is set at a UIC of 150 μg/L.

The Shapiro–Wilk test showed a significant departure of the data from normality: *W* = 0.8572, *P* < 0.001, median 67.7 μg/L, standard deviation 82.79. The median UIC of 67.7 μg/L suggests that the cohort is iodine deficient (Fig. 1). The minimal level was 2.79 μg/L and the maximal concentration was 296 μg/L. Two patients were taking multivitamins (Supradyn® containing 60 μg of iodine and Vitamin Burgerstein® containing 150 μg of iodine) at the time of the UIC measurement. These individuals had UICs of 62.2 μg/L and 135.97 μg/L, respectively. Excluding these two values, the median dropped further to 62.2 μg/L. Of the 39 included patients, 32/39 had a UIC <150 μg/L, 22/39 a UIC <100 μg/L, and 7/39 a UIC ≥150 μg/L (Fig. 2).

There was a significant correlation between BMI and UIC. Twenty-six patients (66.6%) had a BMI <18.5 kg/m^2^, and 13 (33.4%) had a BMI >18.5 kg/m^2^ (*P* < 0.00001); the median UIC in the low BMI group was 57.0 μg/L, and it was 133.3 μg/L in the group with a BMI >18.5 kg/m^2^ (*P* = 0.0466) (Table 2).

**Table 2:** Results of UIC according to BMI. Data are presented as median (IQR).

	BMI, kg/m^2^		*P*-value*
	<18.5 (*n* = 26)	>18.5 (*n* = 13)	
BMI, kg/m^2^	17.21 (14.98–18.45)	20.08 (18.74–23.54)	<0.001
UIC, μg/L	57.03 (2.79–269.18)	133.29 (24.49–296.40)	0.047

IQR, interquartile range; BMI, body mass index; UIC, urinary iodine concentration.

* Statistical comparison by Mann–Whitney.

The median UIC was lower in the purging group compared to the restrictive group. The observation suggests that subjects with purging anorexia are more vulnerable to present with ID, but the difference is not statistically significant. There were no significant differences in BMI between the two groups (Table 3).

**Table 3:** Comparison between purging and restrictive anorexia. Data are presented as median (IQR).

	Purging anorexia (*n* = 11)	Restrictive anorexia (*n* = 28)	*P*-value*
UIC, μg/L	62.20 (17.79–296.40)	96.24 (2.79–290.12)	0.95
Weight, kg	46.40 (40.45–62.45)	49.18 (39.00–66.45)	0.596
Height, cm	164.00 (157.00–178.00)	164.25 (151.50–174.50)	1.0
BMI, kg/m^2^	17.31 (14.98–21.74)	17.82 (14.86–23.54)	0.607

IQR, interquartile range; BMI, body mass index; UIC, urinary iodine concentration.

* Statistical comparison by Mann–Whitney test.

Only 11 of 39 patients (28.2%) had a TSH documented in the electronic medical record. The median TSH was 1.67 mU/L (range: 0.24–2.42 mU/L). None of the patients in whom TSH levels were available had a suppressed or elevated concentration. Due to the paucity of data, it was not possible to determine whether some of the patients had a low T3 syndrome. Thyroglobulin levels were not available for these patients.

## Discussion

The UIC data of the 39 enrolled patients with anorexia nervosa suggest that patients with anorexia nervosa are, as a group, iodine deficient (Figs 1 and 2). In this cohort, the median UIC was 67.7 μg/L. For comparison, the most recent UIC data for Switzerland come from a nationwide cross-sectional study conducted between 2020 and 2022, with results published in 2024. The median UIC was 127 μg/L in children (*n* = 362) and 97 μg/L in pregnant women (*n* = 473) (22). In an earlier nationwide cross-sectional study conducted in 2015–2016, the median UIC in women of reproductive age (*n* = 353) was 88 μg/L, and in pregnant women (*n* = 363) it was 140 μg/L (23). Data from 2014 documented a median UIC of 128.2 μg/L in women (*n* = 715) with a mean BMI of 24.7 kg/m2, and a median UIC of 171.1 μg/L in men (*n* = 705; mean BMI 26.1 kg/m2) (24). Collectively, the median UIC in these studies indicates iodine sufficiency in the Swiss population, and they support the notion that the individuals suffering from anorexia from Switzerland included in this study are iodine deficient (median UIC 67.7 μg/L).

Subjects with lower BMIs had a significantly lower UIC of 57.03 μg/L compared to individuals with a BMI ≥18.5 kg/m^2^ (133.29 μg/L). This observation suggests the presence of a positive correlation between BMI and the severity of nutritional ID. Presumably, this difference indicates a less severe form of the eating disorder in patients with a BMI ≥18.5 kg/m^2^.

The type of anorexia nervosa seems to be an important modifier because patients with binge eating/purging behaviors seem to have more severe ID compared to the group with restrictive anorexia, despite the absence of significant differences in BMI (Table 3). This is consistent with more severe disturbances for other abnormalities in patients with purgative behaviors such as hypokalemia and hyponatremia (25). This may be a consequence of vomiting and/or the use of laxatives in the purgative form, but we have not formally addressed this possibility in our questionnaire.

The strength of this study consists in the fact that this is a prospective study including patients with anorexia followed at two specialized services at a tertiary center. Patients with anorexia nervosa have multiple nutritional deficiencies because of food restrictions that may be further aggravated by purgative behaviors, and, to the best of our knowledge, data on nutritional iodine intake have not been published. Second, the UIC levels have been determined with a highly accurate ion-chromatography mass spectrometry technique that is considered the gold standard, and the measurements were performed in duplicate.

However, this study has several limitations. First, it is based on a small number of samples, given considerations that approximately 125 spot urine samples may be required to estimate the iodine level in a population with 95% confidence and a precision range of ±10% (26). Moreover, the samples have been collected in a tertiary center, which may have led to a selection bias. Second, the samples have been collected at different time points, which may affect the UIC, e.g. a fasting sample may be lower compared to a sample collected after a meal containing foods with a high iodine concentration. Although the samples were immediately stored at 4°C after collection and then frozen as quickly as possible, differences in the handling of these spot urines may have introduced some variability, although urinary iodine is relatively stable. Finally, the current analysis is based on UIC without correction for variability in fluid intake and/or urinary creatinine. High or low fluid intake can influence UIC and potentially lead to an underestimate or overestimate at the individual level. In patients with anorexia, the serum creatinine tends to be low due to the loss in lean body mass; therefore, an eventual correction for urinary creatinine would need to be correlated with serum creatinine. Finally, data on thyroglobulin levels and thyroid function tests are not available.

In summary, the findings presented here are consistent with the initial hypothesis that patients with anorexia nervosa tend to have ID. This, per se, is not surprising given the fact that these patients typically have an unbalanced diet that is not only deficient in calories but also in micronutrients and vitamins. Therefore, patients with anorexia nervosa should receive a multivitamin supplement that contains the recommended daily allowance of potassium iodide. Health care providers involved in the care of these patients should be aware of the need to include an iodide-containing supplement, and the fact that many supplements contain no or insufficient amounts of iodide (27, 28).

To what extent the low UIC contributes to altered thyroid function tests cannot be answered in a definitive way because we do not have sufficient biochemical data on parameters characterizing the thyroid axis. Finally, longitudinal measurements of UIC may be a useful marker to assess whether the patients are compliant with the intake of prescribed supplements. The findings presented here need to be confirmed by larger, representative studies, but further expand the spectrum of the somatic consequences in patients with anorexia nervosa, a prevalent psychiatric eating disorder.

## Supplementary materials



## Declaration of interest

The authors declare that there is no conflict of interest that could be perceived as prejudicing the impartiality of the work reported.

## Funding

The work has been partially supported by unrestricted funds from the Faculty of Biology and Medicine of the University of Lausanne to PAK.

## Author contribution statement

SS and PAK conceived the study, recruited the patients, analyzed the data, and wrote the manuscript. MH was involved in aliquoting urine samples and creatinine measurements. SG provided psychiatric expertise on anorexia nervosa. MBZ supervised the laboratory analyses. MH, SG, and MBZ commented on the form and content of the manuscript.
