# Supplementary material for: Nutritional iodine intake in patients with anorexia nervosa
Source: Eur Thyroid J. 2025 Sep 19;14(5):e250076. doi: 10.1530/ETJ-25-0076 (PMC12450617; doi:10.1530/ETJ-25-0076)
Supplement: Supplementary file 1 [file supplementary_materials.pdf]

Appendix I

Case Report Form

Nutritional iodine intake in patients with anorexia nervosa

Patient Information

Family name	
First name	
Date of birth	
Sex	
Medical record number	

Enrolment

Date of informed consent	
Consent obtained by	
With/without parental consent	

Anthropometric Data

Date	
Height (cm)	
Weight (kg)	
Body Mass Index (kg/m^2)	
Pulse (bpm)	
Blood pressure (mmHg)	

Medical history

Personal medical history	
Family history	

Medication at Enrolment

Medication after Enrolment

Laboratory Data

**Date**	
TSH (mU/l)	
Free T4 (pmol/l)	
Free T3 (pmol/l)	
Anti-TPO antibodies (U/l)	
Anti-TG (U/l)	
Thyroglobulin (ng/ml)	
